# The wasp venom antimicrobial peptide polybia‐CP and its synthetic derivatives display antiplasmodial and anticancer properties

**DOI:** 10.1002/btm2.10167

**Published:** 2020-06-05

**Authors:** Marcelo D. T. Torres, Adriana F. Silva, Gislaine P. Andrade, Cibele N. Pedron, Giselle Cerchiaro, Anderson O. Ribeiro, Vani X. Oliveira, Cesar de la Fuente‐Nunez

**Affiliations:** ^1^ Machine Biology Group, Departments of Psychiatry and Microbiology, Institute for Biomedical Informatics, Institute for Translational Medicine and Therapeutics, Perelman School of Medicine, Penn Institute for Computational Science, and Department of Bioengineering University of Pennsylvania Philadelphia Pennsylvania USA; ^2^ Centro de Ciências Naturais e Humanas Universidade Federal do ABC Santo André SP Brazil; ^3^ Departamento de Bioquímica Universidade Federal de São Paulo São Paulo SP Brazil; ^4^ Departamento de Biofísica Universidade Federal de São Paulo São Paulo SP Brazil

**Keywords:** antimicrobial peptides, Polybia‐CP, rational design, structure‐guided design, wasp venom peptide

## Abstract

The wasp venom‐derived antimicrobial peptide polybia‐CP has been previously shown to exhibit potent antimicrobial activity, but it is also highly toxic. Previously, using a physicochemical‐guided peptide design strategy, we reversed its toxicity while preserving and even enhancing its antibacterial properties. Here, we report on several additional unanticipated biological properties of polybia‐CP and derivatives, namely their ability to target *Plasmodium sporozoites* and cancer cells. We leverage a physicochemical‐guided approach to identify features that operate as functional hotspots making these peptides viable antiplasmodial and anticancer agents. Helical content and net positive charge are identified as key structural and physicochemical determinants for antiplasmodial activity. In addition to helicity and net charge, hydrophobicity‐related properties of polybia‐CP and derivatives were found to be equally critical to target cancer cells. We demonstrate that by tuning these physicochemical parameters, it is possible to design synthetic peptides with enhanced submicromolar antiplasmodial potency and micromolar anticancer activity. This study reveals novel and previously undescribed functions for Polybia‐CP and analogs. Additionally, we demonstrate that a physicochemical‐guided rational design strategy can be used for identifying functional hotspots in peptide molecules and for tuning structure–function to generate novel and potent new‐to‐nature therapies.

## Graphical abstract

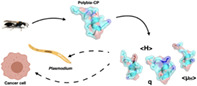

## 1 INTRODUCTION

2

The role of peptides as antimicrobial agents has been extensively described in the literature.^1^, ^2^, ^3^, ^4^, ^5^ New strategies for the design and development of these molecules,^6^, ^7^, ^8^ combined with the rising resistance of microorganisms to standard antibiotics,^9^, ^10^ are boosting worldwide interest and studies on antimicrobial peptides (AMPs). For example, recent reports have described the design of AMPs with broad‐spectrum activity, particularly amphipathic cationic peptides.^11^ Exploring the multifunctional properties of these molecules may lead to candidate molecules that simultaneously kill resistant microorganisms, viruses,^12^ parasite infections,^13^, ^14^, ^15^, ^16^ and cancer cells.^17^, ^18^

Torres et al.^6^ using a physicochemical feature‐guided design of polybia‐CP (Pol‐CP‐NH_2_: Ile‐Leu‐Gly‐Thr‐Ile‐Leu‐Gly‐Leu‐Leu‐Lys‐Ser‐Leu‐NH_2_), identified functional determinants that were key for converting a toxic wasp venom peptide into nontoxic variants with enhanced antimicrobial activity against fungi, Gram‐positive and Gram‐negative bacteria by destabilizing the membrane of those microorganisms. The structure‐guided design of active derivatives of Pol‐CP‐NH_2_ involved reprogramming peptide features to favor the interaction between AMPs and negatively charged biomembranes.

Here, we describe the previously unrecognized ability of Pol‐CP‐NH_2_ and analogs to also target the malaria parasite and cancer cells. These results are significant, as malaria is among the deadliest parasitic infectious diseases according to the World Health Organization, threatening the lives of approximately half of the world's population. Pregnant women and children under 5 years of age are the most common victims of this disease.^19^ Currently, there are limited treatment options available for earlier stages of the disease^20^ and most of the strains are resistant to standard antibiotics. Thus, the best alternative for treating malaria involves preventing infection and monitoring relevant vectors. The anticancer activity of the peptide is also highly relevant, as cancer is a major public health problem worldwide and the second leading cause of death in the United States.^21^ Alternatives for cancer treatment have been sought over the last decades, but effective broad‐spectrum methods have not been reported.

**FIGURE 1:**
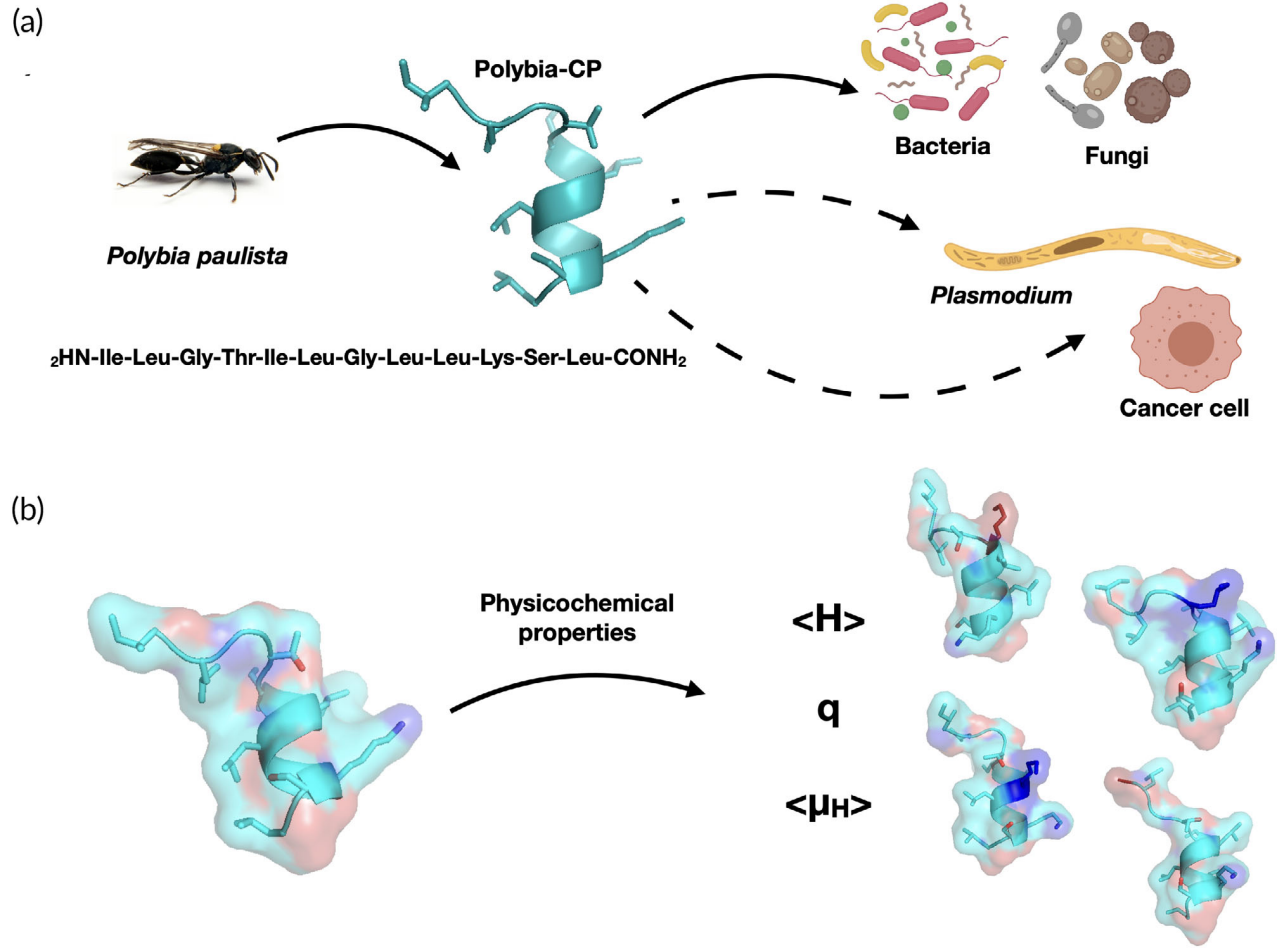
Schematic of the naturally occurring wasp venom peptide polybia‐CP with (a) antimicrobial and prospective antiplasmodial and anticancer activities, by (b) tuning physicochemical features responsible for peptide–membrane interactions

To identify the physicochemical determinants driving these biological properties, we synthesized (Supplementary Table 1) and evaluated the effectiveness of the designed synthetic analogs against *Plasmodium sporozoites* and cancer cell lines (Figure 1a). Our results indicate that physicochemical feature optimization aimed at enhancing the targeting of negatively charged membranes such as those of parasites and cancer cells may provide a viable strategy for treating such diseases (Figure 1b).

## 3 RESULTS AND DISCUSSION

Pol‐CP‐NH_2_ is a potent AMP with in vitro and in vivo activity against bacteria and fungi.^6^ Here, we identified additional biological properties of this peptide and its synthetic analogs through their ability to target malarial sporozoites and cancer cells. In order to analyze the potential of Pol‐CP‐NH_2_ as a triple antimicrobial, antiplasmodial and anticancer agent, we leveraged the systematic design approach proposed by Torres et al., where the main physicochemical properties of the peptides were optimized to achieve increased interactions with negatively charged membranes^6^ and minimize potential enzymatic degradation by avoiding certain motifs and amino acid residues that are targeted by proteases present in blood serum. Briefly, here we engineered specific substitutions into the template sequence of Pol‐CP‐NH_2_ in order to elucidate the structure–function relationships underlying biological function. The modifications were rationally proposed by fine‐tuning physicochemical functional determinants commonly responsible for activity against negatively charged membranes, such as hydrophobicity, hydrophobic moment, net positive charge, amphipathicity, and helical propensity.^6^ The substitutions generated identified physicochemical activity determinants that were important for peptide–membrane interactions.

Hydrophobicity and hydrophobic moment effects on the biological activities of the peptides were evaluated through substitutions by Leu and Phe residues. The aliphatic residue Leu was chosen because of its higher propensity for adopting helical structures compared to other aliphatic or aromatic hydrophobic residues.^22^ Leu residues are also common in wasp venom peptide sequences.^23^ Although Phe presents higher hydrophobicity and, in some cases, potentially toxicity toward eukaryotic cells,^24^ its hydrophobicity is not as high as tryptophan. Thus, by introducing Phe into the original aliphatic residues from the hydrophobic face, it is possible to evaluate the effect of the aromatic residue on structure and biological function. Additionally, unlike Trp, Phe residues are not major components of AMPs,^25^ which are typically cytotoxic, and are therefore better candidates for the design of potential therapeutic agents.

The net charge was analyzed by substituting residues on both faces of the amphipathic helical structure by Lys residues that are frequently found in wasp venom peptides.^23^ Lys was chosen instead of Arg due to its superior flexibility and lower propensity in potentially toxic cell penetrating peptides.^26^ Effects exerted by hydrophobicity‐related and charge‐related substitutions to the helical propensity of the peptides were evaluated in parallel, since structure is crucial to the biological activities of peptides.

To assess the antiplasmodial activity of Pol‐CP‐NH_2_ and its derivatives, the molecules were incubated with *Plasmodium gallinaceum* sporozoites. The avian malaria parasite, *P. gallinaceum*, was chosen as the plasmodium model for this study because it presents lower risk and it is highly similar^27^, ^28^ to existing *Plasmodium falciparum* models responsible for human malaria.^29^

**FIGURE 2:**
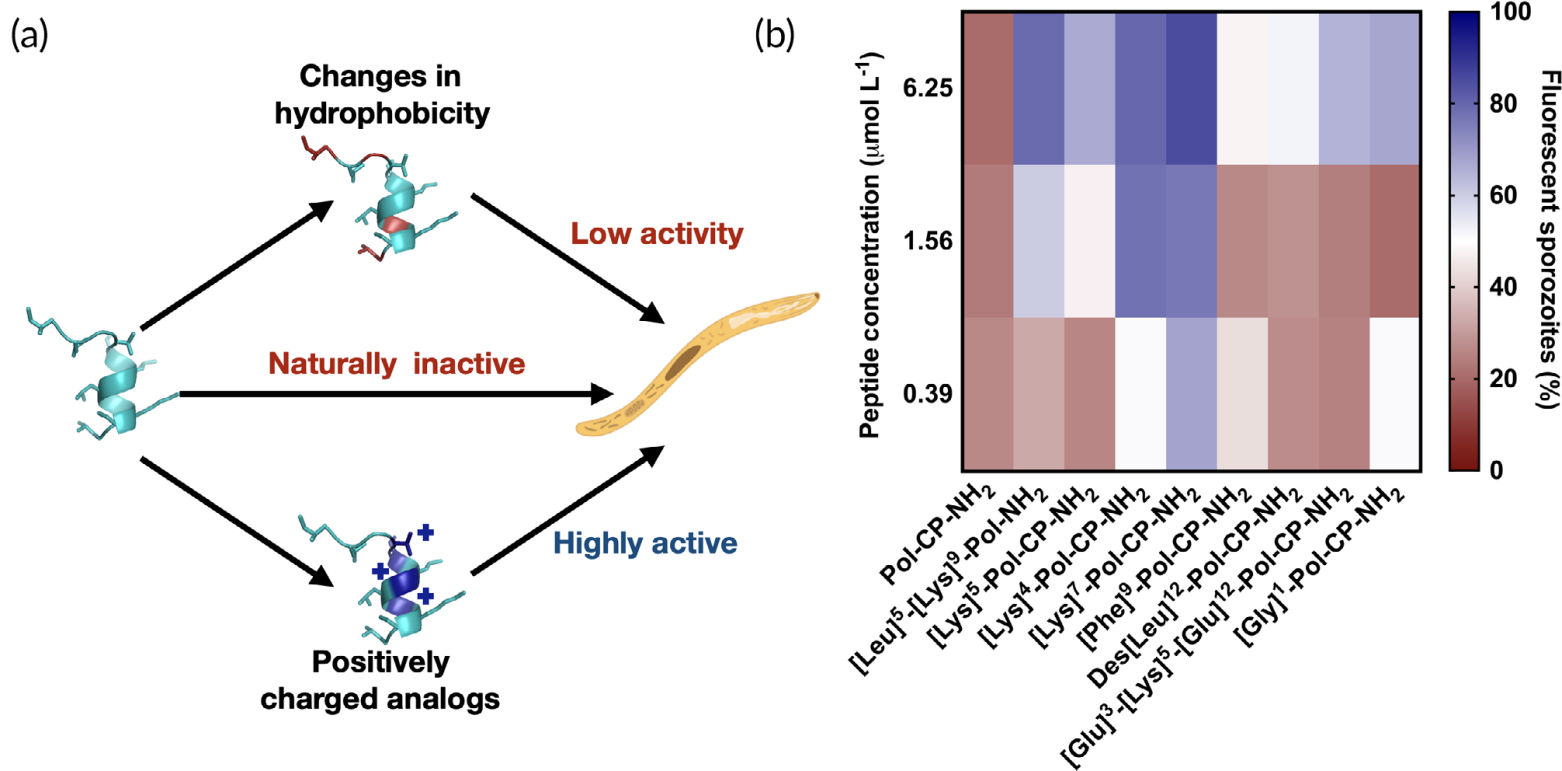
Antiplasmodial activity of polybia‐CP and derivatives in vitro. (a) The importance of physicochemical properties required for antiplasmodial activity exhibited in (b) the heat map containing fluorescent sporozoites (membrane disrupted, shown in blue) in the presence of increasing concentration of the peptides. In red, we highlight the conditions at which the peptides were not active against the *Plasmodium sporozoites*. Experiments were performed in three independent replicates with three repetitions for each condition

The template and designed peptides were screened against *P. sporozoites* in the range of concentrations at which they presented antimicrobial activity against bacteria and fungi (0.39–6.25 μmol L^−1^).^6^ Generally, naturally occurring small cationic peptides that are active against bacteria are not as active against *Plasmodium*.^13^ In fact, Pol‐CP‐NH_2_ did not exhibit antiplasmodial activity at the range of concentrations tested. However, the synthetic peptides designed displaying increased net positive charge showed higher antiplasmodial activity compared to other synthetic peptides described in the literature^13^, ^15^, ^27^, ^28^, ^29^, ^30^, ^31^, ^32^ (Figure 2b). Among the most active analogs, the ones with substitutions within the hydrophilic face and higher helical tendency^6^ presented the highest activity observed, at submicromolar concentrations (Figure 2b). The analogs with the cationic residue Lys substituting Gly in Position 7 and Thr in Position 4 were the most active ones against *Plasmodium*. It has been shown that the addition of positively charged residues in the hydrophilic core of the molecule lead to increased helical content due to stabilization of intramolecular interactions and the well‐defined helical structure of [Lys]^4^‐Pol‐CP‐NH_2_ and [Lys]^7^‐Pol‐CP‐NH_2_ contribute for more effective insertion of the peptides in negatively charged membrane, such as bacteria and *Plasmodium* protozoa, followed by destabilization of the lipid bilayer.^6^ The cationic and helical analogs were even more active against the *Plasmodium* parasite than against bacteria and fungi. At the range of concentrations at which the peptides were active (nanomolar range), they did not exert cytotoxic or hemolytic activities.^6^

Analogs with increased hydrophobicity, which were also the ones with lower helical content values ([Phe]^9^‐Pol‐CP‐NH_2_ and Des[Leu]^12^‐Pol‐CP‐NH_2_), did not present significant antiplasmodial activity at the range of concentrations tested. An important finding during our tests was that peptides with low antimicrobial activity ([Leu]^5^‐[Lys]^9^‐Pol‐CP‐NH_2_ and [Glu]^3^‐[Lys]^5^‐[Glu]^9^‐Pol‐CP‐NH_2_) exhibited antiplasmodial activities, however this only occurred at the higher concentration tested.

**FIGURE 3:**
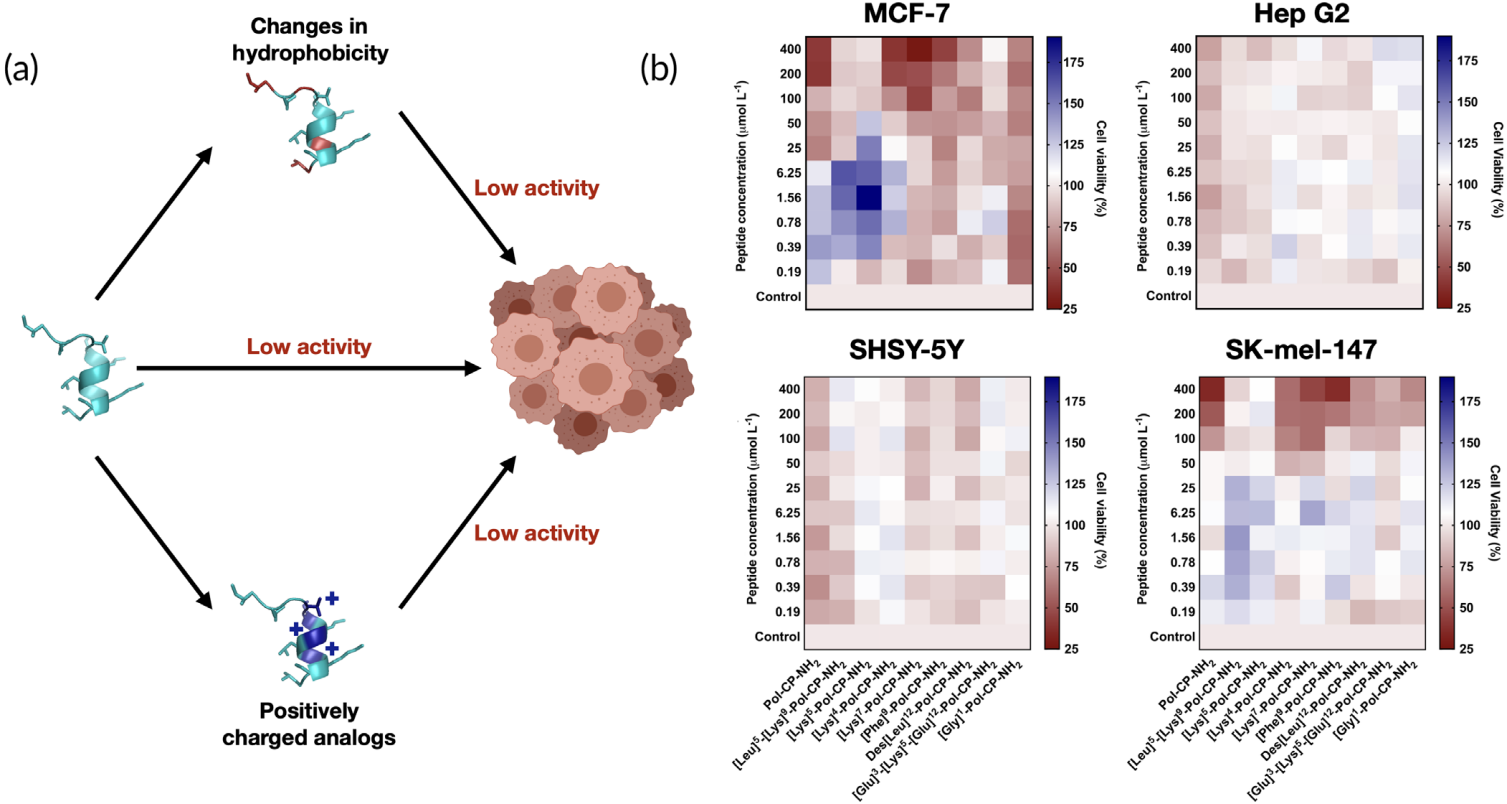
Anticancer activity of polybia‐CP and its derivatives in vitro. (a) The importance of physicochemical properties contributing to anticancer activity exhibited in (b) the heat map containing viable cancer cells exposed to increasing levels of the peptides. In dark red, we show conditions where peptides were active against cancer cell lines. Experiments were performed in three independent replicates with three repetitions for each condition

These results show the viability of our structure‐guided design method for understanding and generating mastoparan‐like peptides with antiplasmodial activity, in this case through tuning of net charge and helicity. Considering the potential of Pol‐CP‐NH_2_ and its analogs as antibacterial, antifungal and antiplasmodial agents, we decided to explore this family of peptides as anticancer agents. In order to assess the anticancer activity of these molecules, we tested them against four different cell lines, human mammary cells (MCF‐7), carcinoma cells of human liver (HepG2), human melanoma (SK‐Mel) cells and neuroblastoma cell (SH‐SY5Y). These cells present different membrane morphology and composition,^33^, ^34^ compared to the healthy mammalian cells, however they all have an abnormal net negative charge^35^ (Figure 3a), because of the overexpression of anionic molecules such as phosphatidylserines, glycoproteins, and glycosaminoglycans,^18^ which we reasoned would interact with our peptides electrostatically.

The inhibitory activities observed against cancer cell lines are at the same range of concentrations reported for antimicrobial activity^6^ (Figure 2b) for this family of peptides. The model molecule, Pol‐CP‐NH_2_, exhibited the highest anticancer activity among all tested peptides (Figure 3b and Supplementary Table 2). It is worth noting that this peptide has no antiplasmodial activity and it is one of the most toxic AMPs of the polybia‐CP family against human embryonic kidney (HEK293) cells at 25 μmol L^−1^.^6^

Generally, the peptides showed increased activity against epithelial‐like cancer cells (MCF‐7 and SK‐Mel) than against neuroblastoma and hepatocyte cells. There is high morphological and physiological heterogeneity between these cells,^36^ making it difficult to elucidate the precise mechanism by which these peptides are more active against certain cell types. However, it is well established that most amphipathic, cationic and helical peptides act on the membranes of cancer cells by electrostatic interactions, subsequently leading to potential internalization into the cell and apoptosis.^37^ Consistent with this notion, increased anticancer effects were observed for those molecules that were more positively charged. Peptides with higher hydrophobicity‐related properties were as active as the wild type and the positively charged analogs against Hep G2 and SHSY‐5Y. The peptides tested in this study presented intermediary activity when compared to other AMPs families. Pol‐CP‐NH_2_ was more active against the cancer cell lines used in this study than VmCT1 and analogs^18^ but it was not as active as decoralin and its derivatives.^17^

Here, we identify novel and previously undisclosed antiplasmodial and anticancer properties for a class of wasp venom‐derived peptides and use a physicochemical feature‐guided design approach to identify relevant functional determinants. Insights derived from such studies provide useful information to build synthetic derivatives with activity against the *Plasmodium* parasite and cancer cell lines. We envision that the principles and approaches exploited here can be applied to other structure–activity studies in order to expand the spectrum of activity of such promising molecules.

## 4 CONCLUSION

AMPs represent promising alternatives to conventional therapies to combat a number of global health problems, including antibiotic resistance,^5^, ^11^ neglected infectious diseases, and cancer. However, the development of AMPs has been limited by the lack of methods for cost‐effective^38^ and rational^39^ design. Although some alternative methods to overcome these limitations have been proposed,^2^, ^3^ we are far from understanding the structure–activity relationship (SAR) of these agents, which would provide a more substantial basis for their rational design and accelerate their translation into the clinic.

In this study, we leveraged a technique involving the structure‐guided‐design of peptides to understand and expand the repertoire of activities of these agents to include antiplasmodial and anticancer properties. Pol‐CP‐NH_2_ and analogs designed to interact with negatively charged biomembranes were shown to be potent antiplasmodial peptides when helical structures were favored by positively charged residues. The peptides tested presented activity against *P. gallinaceum* at submicromolar concentrations. Several peptides with high helical content and increased net positive charge were also active, and in some cases, more active than the wild type, especially at lower concentrations (<25 μmol L^−1^) against epithelial cancer cells. Peptides with higher hydrophobicity than the wild type, as well as Pol‐CP‐NH_2_ and the analogs with higher net positive charge, slightly inhibited the growth of Hep G2 and SHSY‐5Y.

## 5 EXPERIMENTAL PROCEDURES

### 5.1 Solid‐phase peptide synthesis (SPPS), purification, and analysis

Peptides were synthesized on a peptide synthesizer (PS3—Sync Technologies) using the Fluorenylmethyloxycarbonyl (Fmoc) strategy in a Rink Amide resin (substitution degree of 0.52 mmol g^−1^). Procedures for synthesis, purification, analyses, and characterization are described in details by Torres et al.^40^, ^41^

### 5.2 Mosquito rearing and maintenance of the parasite life cycle

*Aedes aegypti* RED strain was used in experiments due to their hypersensitivity to *P. gallinaceum* parasite. Mosquitoes were reared using standard laboratory procedures.^13^, ^17^ An aliquot of frozen chicken blood infected with the *P. gallinaceum* strain 8A was obtained from A Krettli (René Rachou Institute of Research, FIOCRUZ, MG, Brazil). This sample was used to inoculate and establish initial infections in chickens. All subsequent infections of chickens and mosquitoes were accomplished by feeding the mosquitoes on the chickens.

### 5.3 Effect of peptides on salivary gland‐derived *P. gallinaceum* sporozoites

Nine thousand *P. gallinaceum* mature sporozoites were pulled from the salivary glands of *A. aegypti* and incubated in 50 μl of PBS solution, with 40 μmol L^−1^ digitonin (positive control), 0.39–6.25 μmol L^−1^ peptides or negative control (PBS solution), at 37°C for 1 hr. Cell membrane integrity was then observed using a Carl Zeiss inverted fluorescence microscope (model Observer Axio Vision A.1) coupled to an image capture Zeiss AxioCam HR digital camera (1,300 × 1,030 pixels resolution and 8‐bit quantization) after addition of 1 μl of the propidium iodide aqueous solution (200 μmol L^−1^) in 5 μl of total solution volume. Images were obtained using a ×40 objective lens and a green filter effect in red. The spectral range was set with the excitation at 538 nm within the visible spectrum in order to produce orange‐red fluorescence centered at 619 nm, which was processed using the Axio 4.7 software.

### 5.4 Cell culture and treatment

Human mammary adenocarcinoma (MCF‐7) cells were maintained in RPMI 1640 medium supplemented with 10% heat inactivated fetal bovine serum and 100 μg ml^−1^ penicillin/10 μg ml^−1^ streptomycin. While hepatocellular carcinoma (Hep G2) cells, neuroblastoma (SHSY‐5Y) cells and melanoma (SK‐mel‐147) cells were maintained in RPMI 1640 medium supplemented with 10% heat inactivated fetal bovine serum and 100 μg ml^−1^ penicillin/10 μg ml^−1^ streptomycin. One day before the assays, the cells were plated in 96‐well microtiter plate with a density of 2.0 × 10^4^ cells/well at 37°C and 5% CO_2_. On the next day, cells were treated with peptides serial dilutions (0.09–50 μmol L^−1^), incubated in individual microtiter plates for 2 and 24 hr and MTT assays were performed after treatment. Human breast epithelial cells MCF‐10A (ATCC) were maintained in mixture of Dulbecco's modified Eagle's medium and Ham's F12 nutrient mixture supplemented with 5% inactivated horse serum, 10 μg ml^−1^ insulin, 0.02 μg ml^−1^ human epidermal growth factor, 0.5 μg ml^−1^ hydrocortisone, 0.10 μg ml^−1^ choleric toxin, 100 U ml^−1^ penicillin, and 100 μg ml^−1^ streptomycin. The cells were preincubated for 24 hr, plated in 96‐well micro titer plate with a density of 2.0 × 10^4^ cells/well at 37°C and 5% CO_2_. On the next day, cells were treated with peptides serial dilutions (25–100 μmol L^−1^), incubated in individual micro titer plates for 4 and 24 hr and MTT assay was performed after treatment. Experiments were performed in triplicate and all cells were obtained from American Type Culture Collection.

### 5.5 MTT assay

Briefly, MTT (Sigma–Aldrich) was dissolved in water and filtered to make up a 5 μg ml^−1^ solution. Thirty microliters of this solution were added to all the wells which already contained peptide‐treated cells and kept at 37°C for 45 min. Subsequently, the solution was discarded and replaced with 150 μl/well of DMSO and followed by gentle shaking for 15 min. Finally, the microplates were read on an ELISA reader at 570 nm. Experiments were performed in triplicate.

## DISCLOSURE OF INTERESTS

The authors declare no competing financial interests.

## AUTHOR CONTRIBUTIONS

Marcelo D. T. Torresa, Gislaine P. Andrade, Cibele N. Pedron, and Adriana F. Silva performed the experiments. Marcelo D. T. Torresa, Vani X. Oliveira, and Cesar de la Fuente‐Nunez designed the experiments. Marcelo D. T. Torresa and Cesar de la Fuente‐Nunez wrote the manuscript. Vani X. Oliveira, Giselle Cerchiaro, and Anderson O. Ribeiro revised the manuscript.

## Supporting information


**Data S1** Supporting Information.
