# Supplementary material for: The wasp venom antimicrobial peptide polybia‐CP and its synthetic derivatives display antiplasmodial and anticancer properties
Source: Bioeng Transl Med. 2020 Jun 5;5(3):e10167. doi: 10.1002/btm2.10167 (PMC7510464; doi:10.1002/btm2.10167)
Supplement: Supplementary file 1 — Data S1 Supporting Information. [file BTM2-5-e10167-s001.docx]

**The wasp venom antimicrobial peptide polybia-CP and its synthetic derivatives display antiplasmodial and anticancer properties**

Marcelo D. T. Torres^a^, Adriana F. Silva^b,c^, Gislaine P. Andrade^b^, Cibele N. Pedron^b^, Giselle Cerchiaro^b^, Anderson O. Ribeiro^b^, Vani X. Oliveira Jr^b,d,^* and Cesar de la Fuente-Nunez^a,^*

**Supplementary Material**

**Supplementary Table 1. Characterization of Pol-CP-NH_2_ and derivatives.**

| **Peptide** | **HPLC Purity^a^** | **Molecular Weight (Da)** | **(M + H^+^) (Da)^b^** |
| --- | --- | --- | --- |
| Pol-CP-NH_2_ | 99% | 1239.8 | 1241 |
| [Leu]^5^-[Lys]^9^-Pol-CP-NH_2_ | 99% | 1254.8 | 1256 |
| [Lys]^5^-Pol-CP-NH_2_ | 99% | 1254.8 | 1256 |
| [Lys]^4^-Pol-CP-NH_2_ | 98% | 1265.8 | 1267 |
| [Lys]^7^-Pol-CP-NH_2_ | 99% | 1309.8 | 1310 |
| [Phe]^9^-Pol-CP-NH_2_ | 99% | 1272.8 | 1274 |
| Des[Leu]^12^-Pol-CP-NH_2_ | 99% | 1125.8 | 1127 |
| [Glu]^3^-[Lys]^5^-[Glu]^12^-Pol-CP-NH_2_ | 99% | 1341.8 | 1342 |
| [Gly]^1^-Pol-CP-NH_2_ | 99% | 1182.8 | 1184 |

^a^HPLC profiles were obtained under the following conditions: Column Supelcosil C_18_ (4.6 x 150 mm), 60 Å, 5 μm; Solvent System: A (0.1% TFA/H_2_O) and B (0.1% TFA in 60% ACN/H_2_O); Gradient: 5–95 % B in 30 minutes; Flow: 1.0 mL min^-1^; λ = 220 nm; Injection Volume: 50 μL and Sample Concentration: 1.0 mg mL^-1^.

^b^LC/ESI-MS data were obtained on a Model 6130 Infinity mass spectrometer coupled to a Model 1260 HPLC system (Agilent), using a PhenomenexGemini C_18_ column (2.0 mm x 150 mm, 3.0 μm particles, 110 Å pores). Solvent A was 0.1% TFA in water, and solvent B was 90% ACN in solvent A. Elution with 5–95% B gradient was performed over 20 min, 0.2 mL min^-1^ flow and peptides were detected at 220 nm. Mass measurements were performed in a positive mode with the following conditions: mass range between 100 to 2500 m/z, ion energy of 5.0 V, nitrogen gas flow of 12 L min^-1^, solvent heater of 250 ^o^C, multiplier of 1.0, capillary of 3.0 kV and cone voltage of 35 V.

**Supplementary Table 2. Concentration of peptide needed for killing 50% (IC_50_) of four different cancer cell lines:** human mammary cells (MCF-7), carcinoma cells of human liver (HepG2), human melanoma (SK-Mel) cells and neuroblastoma cell (SH-SY5Y)**.**

| **Peptide** | **IC_50_ (μmol L^-1^)** | | | |
| --- | --- | --- | --- | --- |
|  | **MCF-7** | **HepG2** | **SK-mel-147** | **SH-SY5Y** |
| Pol-CP-NH_2_ | 200 | NO | 200 | NO |
| [Leu]^5^-[Lys]^9^-Pol-CP-NH_2_ | NO | NO | NO | NO |
| [Lys]^5^-Pol-CP-NH_2_ | NO | NO | NO | NO |
| [Lys]^4^-Pol-CP-NH_2_ | 200 | NO | 100 | NO |
| [Lys]^7^-Pol-CP-NH_2_ | 100 | NO | 100 | NO |
| [Phe]^9^-Pol-CP-NH_2_ | 400 | NO | 200 | NO |
| Des[Leu]^12^-Pol-CP-NH_2_ | NO | NO | NO | NO |
| [Glu]^3^-[Lys]^5^-[Glu]^12^-Pol-CP-NH_2_ | NO | NO | NO | NO |
| [Gly]^1^-Pol-CP-NH_2_ | NO | NO | NO | NO |

NO, not observed.
